# Is Culture Important to the Relationship Between Quality of Life and Resilience? Global Implications for Preparing Communities for Environmental and Health Disasters

**DOI:** 10.3389/fpsyg.2020.01492

**Published:** 2020-07-30

**Authors:** Suzanne M. Skevington, The WHOQOL SRPB Group, M. Bartos

**Affiliations:** Melbourne, Australia; La Plata, Argentina; Porto Alegre, Brazil; Guangzhou, China; Alexandria, Egypt; Chennai, India; Pondicherry, India; Beersheba, Israel; Rome, Italy; Tokyo, Japan; Eldoret, Kenya; Vilnius, Lithuania; Kubang, Malaysia; Barcelona, Spain; Umeå, Sweden; Bangkok, Thailand; İzmir, Turkey; Bath, United Kingdom; Montevideo, Uruguay; At WHO Geneva: United States.; Manchester Centre for Health Psychology, Division of Psychological Science and Mental Health, The University of Manchester, Manchester, United Kingdom

**Keywords:** resilience, quality of life, culture, model, health, adversity, environment

## Abstract

**Background:**

Using a preventative approach, we investigated whether international subjective qualities of life are associated with resilience to adversity when culture is taken into account. Although resilience has been previously associated with good QoL, cross-cultural studies are scarce.

**Methods:**

Sequential linear multiple regression models of WHOQOL SRPB data from 15 countries worldwide (*N* = 3,019) examined which qualities are most closely associated with resilience, when adjusting for culture and selected demographics. We also examined whether all cultures confirmed this positive association. Of 13 QoL facets identified from a literature summary, seven were associated with defining resilience and six reflected strategies for building resilience; these were tested together. Principal components analysis provided a dependent variable for resilience, covering inner strength and hope.

**Results:**

The final model explained 52% of resilience overall, of which QoL explained 37% and culture explained 12% (*p* < 0.0001). Being older than 45 years was a significant covariate. Spiritual QoL from meaning in life, awe and wonder, wholeness and integration, and being kind to others was linked with strategies for building resilience (28%). Better psychological QoL from high levels of positive feelings and low negative feelings was associated with defining resilience (9%). Larger significant positive β’s were found for 10 cultures, so model “universality” was not confirmed.

**Conclusion:**

A new cross-cultural psycho-spiritual model of resilience is presented. Assessing individual QoL profiles could identify suitable community members to build resilience locally in culturally acceptable styles. The WHOQOL SRPB evidence could inform international policy designed to prepare vulnerable cultures that are threatened with environmental and health disasters.

## Introduction

The universal mechanisms for building psychological resilience are not well understood, but observing human response to natural disasters illustrates how vulnerable communities, individuals, and systems can improve their resilience (Rodin, 2014). Community investment in building psychological resilience before a disaster has resulted in better physical health and less psycho-social trauma. Being ready and responsive to deal with adversity increases the capacity to survive and adapt and grow from shocks (American Psychological Association [APA], 2014). Resilient people appear to “bounce back” more rapidly and emerge stronger, so investing in preparation pays a “resilience dividend” (Rodin, 2014). Furthermore, geographical studies show that socio-economically deprived communities tend to live in high-risk locations for physical hazards (Curtis, 2004), so research on building resilience can prepare poorer people for environmental threats. A preventative approach to disaster management is an integral part of protecting human planetary health (Whitmee et al., 2015). To achieve international sustainable development goals (SDGs) by 2030 will require increasing resilience to adversity at the individual, community, cultural, city, organizational, institutional, and network levels (United Nations Strategy for Disaster Reduction [UNSDR], 2009), so empirical evidence will be necessary to inform this initiative.

Evidence increasingly shows that cultural communities are sustained by developing resilience (e.g., Ungar, 2008). In a major summary of the findings from resilience research (American Psychological Association [APA], 2014), many studies were conducted in a single Western culture. Furthermore, the choice of variables assessed within them suggested a predominantly psycho-social model. Although research has linked resilience to well-being and QoL (e.g., Bottolfs et al., 2020), it is not known whether this model is “universal.” Such information could be valuable to vulnerable cultures and also to policy-makers seeking to prepare local communities for disaster, e.g., from climate change or pandemic. The availability of international data and methods offers an opportunity for original research.

Progress in this field has been impeded by a shortage of measures that enable the accurate cross-cultural comparisons of subjective QoL to be made across many different cultures. In recent years, the WHOQOL suite of multi-lingual and multi-dimensional measures has improved the QoL comparisons by applying a new methodology and broadening the contents to include the largely neglected domains of environmental QoL (Lercher, 2003) and spiritual QoL (see Skevington et al., 2004). Among many health and well-being studies relevant to the present work, the WHOQOL-BREF has been widely used to assess vulnerable communities living in impoverished “high-risk” environments. These include slums (Skevington, 2009; Simonelli et al., 2013), refugee camps (Akinyemi et al., 2012), earthquake zones (Ardalan et al., 2011; Guo et al., 2012; Valenti et al., 2013), radiation sites (Yen et al., 2013), political conflict areas (Hammoudeh et al., 2013), and torture victims (Pabilonia et al., 2010). This body of research illustrates how adversity affects QoL in very diverse cultures, and this type of evidence could assist in delivering an appropriate humanitarian response.

In a further application, the present study seeks to examine the relationship between QoL and resilience in culturally diverse communities; such findings could support preparations for catastrophic events. Furthermore, in a summary of research findings on resilience (American Psychological Association [APA], 2014), qualities of life were implicitly linked to factors that define resilience and strategies for building resilience (e.g., Grant and Kinman, 2012), and our international dataset was used to investigate these QoL clusters.

The World Health Organization defines subjective QoL as “an individual’s perception of their position in life, in the context of the culture and value systems in which they live, and in relation to their goals, expectations, standards and concerns” (WHOQOL Group, 1994). This culture-focused definition underpins the current research.

In the present cross-cultural study, we aimed to investigate whether culture influences the association between subjective QoL and resilience to adversity. We hypothesized that culture would have a significant impact on this significant positive association. In addition, we examined the “universality” of the association by assessing it in individual cultures, where large significant betas (β) were hypothesized. Furthermore, we hypothesize that, based on previous findings, specified clusters of QoL facets would be associated with defining resilience and with practical strategies to build it. If cross-cultural data confirmed the findings about the qualities of life being associated with building resilience, this could inform “high-risk” communities who are making preparations to face challenging environmental or health events and assist in the decisions of international policy-makers.

## Materials and Methods

### Sampling and Procedures

A cross-sectional, general population survey was conducted contemporaneously in 15 countries worldwide. Each national center targeted 240 adults. A quota sampling design was applied (2 × 2), and convenience sampling was used within quotas for gender (50% women and men) and age band (50% over/under 45 years) (WHOQOL SRPB Group, 2006). The national centers are located in South America [Argentina: La Plata (*n* = 225); Brazil: Porto Alegre (*n* = 253); Uruguay: Calabria (*n* = 250)], Africa [Kenya: Eldoret (*n* = 240)], Asia [India: Bangalore (*n* = 241); Japan: Tokyo (*n* = 44); Malaysia: Kubang (*n* = 240); Thailand: Bangkok (*n* = 188)], Middle East [Egypt: Alexandria (*n* = 240); Israel: Beersheba (*n* = 267); Turkey: İzmir (*n* = 225)], Southern Europe [Italy: Rome (*n* = 102); Spain: Barcelona (*n* = 240)], and Northern Europe [Lithuania: Vilnius (*n* = 239): United Kingdom: Bath (*n* = 277)]. Specific profiles of religious, spiritual, and personal beliefs were constructed in each center from national statistics to guide the proportionate sampling locally. The total sample included the following subgroups: Buddhist, Zen Buddhist, Hindu, Muslim, Jewish, Atheist, Christian (Catholic/Protestant), and other belief systems (e.g., animism) (WHOQOL SRPB Group, 2002). Demographic information on culture, gender, highest educational level, age, and subjective health were also recorded.

Following ethical approval from the World Health Organization and the ethics committee in every center, the participants were recruited in a variety of urban, suburban, and rural settings. After gaining informed consent, a self-reported measure of QoL was administered, or it was administered by the interviewers in locations where literacy was low.

The models in the present study included 3,019 adults from 15 countries, with a mean age of 42.5 years. Of these participants, 27.4% were women <45 years and 24.0% were >45 years; 26.3% were younger men and 22.3% were older. The mean education level completed was secondary school (2.3 on a four-point scale, with a slightly positive skew). The mean subjective health was 3.6 (five-point scale). On the day of the survey, 57% said that they were “well.” A total of 43% reported one or more chronic diseases or conditions. The primary health problems included blood pressure (7.3%), heart disease (6.6%), emotions (5.4%), arthritis (4.7%), cancer (3.5%), respiratory (3.5%), bone (2.8%), diabetes (2.6%), feet (1.2%), HIV (0.9%), rectal (0.8%), Parkinson’s disease (0.6%), and others (11.7%).

### Measures

#### The World Health Organization Quality of Life Assessment of Spiritual, Religious and Personal Beliefs (WHOQOL SRPB Group, 2006)

The WHOQOL SRPB measures subjective QoL as applied to health. Ninety-two focus groups of patients, health professionals, and community members were convened in 18 national centers (*n* = 701) to offer information about the contents of this cross-cultural person/patient-reported outcome measure. As evidence of resilience, they recorded that QoL gained from inner strength offered stability in life, which helped them face difficulties and overcome adversity. This was derived from cultural traditions, “god,” character, personal philosophy, family/community support, and other external sources (World Health Organisation [WHO], 2000). Multiple language versions of the WHOQOL SRPB were developed simultaneously to standardize the measure in collecting international survey data (WHOQOL SRPB Group, 2006). A novel “spoke–wheel” methodology was devised, whereby the participating cultures agreed a feasible protocol for completing the international work to create a measure. Cultural adaptation and back-translation procedures advanced the semantic and the conceptual equivalence between WHOQOL language versions, thus improving the accuracy of cross-cultural comparisons (Bowden and Fox-Rushby, 2003; Skevington et al., 2004). The WHOQOL has been used to understand responses to biodiversity (United Nations Environment Program [UNEP], 2011; Skevington et al., 2019). If included in international surveys, it could usefully assess, for example, whether SDG well-being targets have been achieved in 2030 (Skevington and Epton, 2018).

The WHOQOL SRPB contains 132 items organized in 33 QoL facets and scored in one of six domains: physical, psychological, independence, social, environmental, and spiritual QoL. A general QoL facet assesses overall QoL with health. The WHOQOL SRPB contains 25 facets (represented by 100 items from the WHOQOL-100) and eight QoL facets (32 additional items) on spiritual, religious, and personal beliefs (SRPB) (WHOQOL SRPB Group, 2006). As the original “spiritual” facet from the WHOQOL-100 has been integrated into the SRPB domain, nine facets are scored. The resilience literature indicates that “kindness to others” is important (American Psychological Association [APA], 2014), so an optional facet covering this topic was also included. Kindness to others was piloted in the WHOQOL SRPB survey but excluded from the final measure; it is appended in the manual with two other facets. The WHOQOL SRPB group permits the inclusion where the context is appropriate, but it is not scored in a domain (WHOQOL SRPB Group, 2002). In summary, a total of 136 items covering 34 facets were assessed.

#### Poverty Indicator

In view of the range of cultures being assessed, we were aware that national poverty levels might explain some of the findings (Skevington, 2009). After reviewing the literature on international poverty indicators, national education was selected from the Human Development Index (HDI) (United Nations Development Program [UNDP], 1990). Furthermore, the HDI values for all participating countries were available for the year of the WHOQOL SRPB survey. These scores range from 0 (highest poverty) to 1.0 (lowest). This indicator is considered a proxy for poverty as it encompasses income, wealth, and social status (Manly, 2006). Direct questioning about poverty is offensive in some cultures.

### Operationalizing Variables

The WHOQOL SRPB contents were reviewed with reference to the summary of resilience research on the American Psychological Association’s website (American Psychological Association [APA], 2014)^1^. Four items that constitute the facet on *inner strength* most closely reflected resilience to adversity. The remaining seven items were drawn from facets on *hope and optimism*, *connection to a spiritual being*, *faith*, and *personal beliefs*. These 11 items provided a pool from which the dependent variable (DV) items were selected.

The APA resilience characteristics were then mapped onto relevant WHOQOL SRPB facets to operationalize them. These 13 facets of QoL were hypothesized to be closely associated with resilience. Within these facets, two conceptual QoL clusters that define resilience or are concerned with practical strategies for building resilience were distinguished. Seven facets (in italics) were linked with defining resilience: (i) making realistic plans necessary for high-level decision-making (*cognitions*), (ii) having sufficient *energy* to implement plans, (iii) having a positive self-image, with good *self-esteem* and self-confidence, (iv) good communication and problem-solving skills, within positive *personal relationships*, (v) *practical social support*, and (vi and vii) strong *positive feelings* and weak *negative feelings.* Four of these facets were taken from the psychological domain, two from the social domain, and one from the physical domain. Six QoL facets were linked with practical strategies for building and maintaining resilience: (viii) opportunities to *acquire new information and skills*, (ix) opportunities to participate in *recreation and leisure*, (x) *meaning in life*, (xi) *awe and wonder*, (xii) *wholeness and integration*, and (xiii) *kindness to others*; this optional facet is rarely researched. Four of the six facets were drawn from the spiritual domain and two from the environment domain.

### Analysis Plan

Secondary analysis was conducted on a subset of data from a cross-sectional survey designed to standardize the WHOQOL SRPB (WHOQOL SRPB Group, 2006). The data were previously used to investigate QoL and well-being (Skevington and Boehnke, 2018) and in relation to biodiversity (Skevington et al., 2019).

Sequential (hierarchical) multiple linear regression analysis (SPSS v22) modeled the impact of culture on the association between QoL and resilience, adjusted for demographic characteristics and health. Although cultures are nested within the data, this statistic was chosen to accommodate a large number of potential predictor variables and permit the identification of those with the greatest predictive power. To build the model, independent variables were entered in blocks so that a complete regression solution could be examined at every stage. First, all demographic and health variables were tested together as potential covariates in an initial model (not shown), with the aim of discarding non-significant variables. Three covariates were then entered into the model at step 1. All cultures were entered together at step 2. Two further blocks containing clusters of QoL IVs were then entered, one containing qualities of life defining resilience (step 3) and, lastly, for practical strategies to build resilience (step 4). Model fit was estimated from delta adjusted *R*-square (Δ*R*^2^), *R*^2^ change, and *F* significance. Betas (β) and *t*-tests with their *p*-values showed how resilience changed in relation to an IV when all other IVs in the model are adjusted. Standardized partial regression coefficients (*r*) were compared. The entry criterion for *F* was *p* = 0.05; removal 1.0. The outliers were identified from normal probability plots of standardized residuals with *z* predictors (>2.0 SDs). Collinearity changes, Eigenvalues (>1.0), Cooks leverage, and Durbin–Watson statistics (<2.0 criterion) were calculated.

*A posteriori* one-tailed Pearson correlations examined whether the association strength between QoL and resilience measured by extracting betas (β) from model 4, for each culture, was related to the adversity of poverty, as assessed by national HDI values.

Where data were randomly missing, the culture mean for that variable was imputed. The cases were deleted where missing data was >20%. Where two or more items are missing (one in the social domain), the WHOQOL domains are not scored. A few variables were mildly skewed [standard deviations (SDs) < 1.0], but as negligible improvement resulted from log transformation, the original scores were retained. Case-wise diagnostics and multivariate outliers informed deletion. Multi-collinearity, singularity, and part/partial correlations facilitated the adjustments (Tabachnick and Fidell, 2006). Power analysis used a high case to IV ratio for a mildly skewed DV to detect a small effect. As a minimum of 1,520 cases were needed, the sample was acceptable.

Multiple categorical (“dummy”) variables for each national center were calculated. In the absence of hypotheses about particular cultures and because the working language of the WHOQOL group is English, the United Kingdom center was designated the reference category (Katz, 2006). Merging adjacent categories in ordinal variables reduced the skew, where the cell numbers were small. Binary variables were created for gender (female/male). For marital status, four categories were combined into “not married” and then compared with “married.” Three educational levels formed “lower education” for comparison with “higher” (tertiary) education. The variables were not analyzed where distributions were poor, if the DV correlated very highly with similar IVs thus duplicating information, and where potential covariates correlated very highly with IVs. Negligible DV correlations with gender and with marital status excluded these covariates.

### Developing a Dependent Variable on Resilience

To provide a resilience DV for modeling, 11 WHOQOL SRPB items from facets on *inner strength* (four items), *spiritual connection* (three items), *purpose in life* (two items), *hope and optimism* (one item), and *faith* (one item) were tested for internal consistency reliability (ICR). Standardized Cronbach’s alpha (α) with item substitution was applied and showed excellent ICR (α = 0.901). All inter-item correlations between candidate items were acceptable (*r* = 0.33 to 0.59).

A principal component analysis (PCA) examined whether it was justifiable to combine all 11 items into one DV or subsets. The Kaiser–Meyer–Olkin test affirmed acceptable multivariate normality. PCA extraction was good (0.886), and Bartlett’s test for sampling adequacy was acceptable (*p* < 0.0001). A scree of Eigenvalues (>1.0) from the unrotated solution identified three factors that were subsequently supported by Varimax rotation (five iterations). Item loadings >0.40 were deemed acceptable.

The final solution explained 72.43% of the total variance. Factor 1 (F1) contained five item loadings (0.57–0.89), accounting for 27.92% of the total. It consisted of four items on *inner strength* (“To what extent can you find spiritual strength in difficult times?,” “How much does spiritual strength help you to live better?,” “To what extent do you feel inner spiritual strength?,” and “To what extent does your spiritual strength help you to feel happy in life?”). One item on *hope and optimism* was added (“How able are you to remain optimistic in times of uncertainty?”). Four other items were loaded onto factor 2 (F2), accounting for 27.01% of the variance. F2 included three items on *spiritual connection* and one on *faith*. In factor 3 (F3), two items from the *spirituality* facet were included on *personal beliefs* (17.49%).

The PCA factor structure showed that a single DV of 11 items was not justified. The five F1 items cohere to provide a meaningful representation of resilience, and together they show good ICR (α = 0.834). The subtotal score of these five items ranged from 5 to 25 (*M* = 16.63; SD = 3.8). Due to the interval properties of the WHOQOL response scales (Skevington and Tucker, 1999), this factor subtotal forms a recognized scale; hence, this score was adopted as the resilience DV in the regression analysis. Furthermore, these scores are more easily interpreted than the factor loadings.

## Results

Across 33 facets, mean QoL was acceptable to good, skew was minor, and kurtosis was slight. The mean general QoL and health was 3.64 (SD 0.9; range 1–5). Domain ICRs range from 0.75 to 0.89. Means (M) and (SDs) for WHOQOL SRPB domains and resilience for the 15 cultures sampled are shown in Table 1. Most resilience means for participating countries were higher than the mid-point (15.0); Malaysia reported the highest resilience (18.4), and Japan reported the lowest (13.9). Overall, the spiritual and environmental QoL domains were the lowest and independence QoL was the highest. Domain means showed that QoL ranged from very good (>70) (independence QoL in Italy) to poor (<50) (spiritual QoL in Japan); the mid-point of 50.0 indicates that QoL is okay. SDs ranged from 21.4 (United Kingdom, physical) to 9.4 (Argentina, spiritual).

**TABLE 1:** Descriptive statistics for resilience and WHOQOL SRPB quality-of-life (QoL) domains in 15 cultures.

Resilience/QoL domain Cultures	Resilience		Physical		Psychological		Independence		Social		Environment		Spiritual	
	*M*	*SD*	*M*	*SD*	*M*	*SD*	*M*	*SD*	*M*	*SD*	*M*	*SD*	*M*	*SD*
Argentina	16.88	1.69	59.74	17.93	53.66	12.74	59.02	17.04	54.01	14.38	47.68	9.68	54.82	9.39
Brazil	18.29	3.84	56.81	15.90	65.55	12.45	67.79	19.93	69.59	13.44	60.06	10.98	68.89	15.02
Egypt	18.32	3.30	49.00	16.42	60.24	15.61	57.76	19.52	61.05	15.68	53.01	13.91	68.97	10.81
India	16.42	4.52	73.07	17.94	71.78	13.82	75.40	17.67	69.23	14.60	67.76	14.79	62.79	16.92
Israel	16.52	4.15	62.84	18.26	68.99	13.78	77.08	19.39	71.41	15.58	66.83	12.17	59.84	15.64
Italy	14.20	3.94	71.96	13.80	66.25	13.06	79.43	11.53	67.24	11.98	65.71	10.10	55.46	15.82
Japan	13.88	4.82	58.37	14.74	54.08	17.11	65.55	13.26	57.09	13.46	56.31	14.62	46.56	19.71
Kenya	14.56	3.48	59.68	15.69	56.34	10.98	66.74	17.80	62.61	13.03	53.78	11.04	49.11	12.80
Lithuania	18.35	3.12	61.76	15.23	68.87	10.94	66.19	16.15	66.61	12.14	63.63	11.20	69.42	12.28
Malaysia	18.38	3.22	56.00	16.34	62.85	12.08	59.40	16.94	64.03	13.64	52.02	13.69	65.24	12.34
Spain	15.93	2.82	60.54	16.10	60.36	13.70	71.10	17.22	62.31	15.95	61.34	10.73	59.00	11.72
Thailand	16.14	3.86	70.22	15.77	69.34	13.33	77.87	12.32	68.93	10.73	65.39	12.59	53.35	12.38
Turkey	15.44	4.55	59.31	15.92	63.14	15.25	66.26	15.48	61.93	16.99	57.54	14.35	56.51	18.00
United Kingdom	15.71	4.18	63.38	16.99	63.64	14.14	71.75	21.57	66.66	16.40	66.64	11.06	58.36	17.13
Uruguay	17.50	3.19	62.62	21.40	59.11	15.11	65.92	19.27	66.80	14.84	58.05	12.17	57.86	14.20
Total sample	16.70	3.87	61.35	17.86	63.36	14.43	68.17	18.89	65.06	15.12	59.74	13.68	60.16	15.47

*WHOQOL = transformed scores 0–100. Resilience scores 5–25. From WHOQOL SRPB Group (2006).*

### Does Subjective QoL Predict Resilience Internationally When Culture Is Included?

In Table 2, 51.6% of the variance in resilience was accounted for by QoL after adjusting the model for culture, demographics, and health (Durbin-Watson 1.9); this result confirms the primary hypothesis. Culture explained 11.6% of the total resilience, confirming the hypothesis that it would make a highly significant contribution (*p* < 0.0001). Culture was one of the four blocks of IVs that significantly and sequentially increased the amount of variance explained (*p* < 0.0001). The QoL cluster that had been previously linked to strategies for building resilience best explained resilience, it accounted for 28.2% of the variance. This contrasted with the 8.8% explained by positive and negative feelings that define resilience. Although self-esteem and cognitions facets were significant in model 3, these facets disappeared from the final model when six other facets were entered in model 4.

**TABLE 2:** Sequential (hierarchical) multiple linear regression analysis models for the association between resilience and quality of life, taking account of culture and demographics.

Variable	Model 1 demographics only				Model 2 demographics + cultures				Model 3 demographics + cultures + defining qualities of life				Model 4 demographics + cultures + defining QoL + strategies for building resilience			
	
	β	*t*	*p*	*r*	β	*t*	*p*	*r*	β	*t*	*p*	*r*	β	*t*	*p*	*r*
**Step 1**																
Older age	0.103	50.63	0.0001	0.10	0.095	50.47	0.0001	0.09	0.083	40.96	0.0001	0.09	0.038	20.89	0.004	0.05
Health	0.175	90.53	0.0001	0.17	0.150	80.13	0.0001	0.15	0.047	20.37	0.018	0.04	–0.013	–0.84	0.399	–0.02
Higher education	–0.031	–10.72	0.085	–0.03	0.054	20.87	0.004	0.05	0.029	10.58	0.113	0.03	0.019	10.35	0.177	0.03
**Step 2**																
Argentina					0.134	50.87	0.0001	0.10	0.197	80.76	0.0001	0.16	0.220	110.39	0.0001	0.20
Brazil					0.181	80.94	0.0001	0.16	0.157	70.99	0.0001	0.14	0.108	60.86	0.0001	0.12
Egypt					0.178	70.97	0.0001	0.14	0.185	80.33	0.0001	0.15	0.152	80.34	0.0001	0.15
India					0.074	30.33	0.001	0.06	0.051	20.35	0.019	0.04	0.045	20.58	0.010	0.05
Israel					0.055	20.42	0.015	0.04	0.027	10.24	0.214	0.02	0.018	10.02	0.307	0.02
Italy					–0.06	–30.17	0.002	–0.06	–0.059	–30.08	0.002	–0.06	–0.030	–20.01	0.045	–0.04
Japan					–0.05	–20.72	0.007	–0.05	–0.021	–10.20	0.232	–0.02	0.019	10.36	0.174	0.03
Kenya					0.200	80.89	0.0001	0.16	0.211	90.55	0.0001	0.17	0.182	100.03	0.0001	0.18
Lithuania					–0.04	–10.75	0.080	–0.03	0.012	0.52	0.604	0.01	0.038	20.11	0.035	0.04
Malaysia					0.211	90.15	0.0001	0.16	0.185	80.21	0.0001	0.15	0.148	80.13	0.0001	0.15
Spain					0.028	10.25	0.211	0.02	0.042	10.97	0.049	0.04	0.030	10.72	0.086	0.03
Thailand					0.040	10.83	0.067	0.03	0.018	0.86	0.392	0.02	0.155	80.34	0.0001	0.15
Turkey					–0.01	–0.33	0.743	–0.01	–0.012	–0.54	0.592	–0.01	0.051	20.93	0.003	0.05
Uruguay					0.131	50.81	0.0001	0.11	0.155	70.15	0.0001	0.13	0.173	90.90	0.0001	0.18
**Step 3**																
Energy									–0.030	–10.32	0.186	–0.02	–0.004	–0.20	0.837	–0.01
Positive Feelings									0.203	80.54	0.0001	0.15	0.066	30.33	0.001	0.06
Cognition									0.058	20.45	0.014	0.05	0.022	10.14	0.253	0.02
Self-esteem									0.109	40.29	0.0001	0.08	0.031	10.51	0.131	0.03
Negative Feelings									–0.010	–0.47	0.637	–0.01	0.036	20.10	0.036	0.04
Personal Relat’ns									0.014	0.57	0.569	0.01	–0.037	–10.91	0.056	–0.04
Social Support									0.055	20.72	0.007	0.05	–0.019	–10.11	0.266	–0.02
**Step 4**																
Information													–0.012	–0.70	0.487	–0.01
Recreation													0.019	10.01	0.310	0.02
Meaning													0.290	170.08	0.0001	0.30
Awe													0.211	110.87	0.0001	0.21
Whole													0.233	140.38	0.0001	0.25
Kindness													0.114	70.74	0.0001	0.14

*R^2^ = 0.035 for Step 1; ΔR^2^ = 0.146 for Step 2; ΔR^2^ = 0.232 for Step 3; ΔR^2^ = 0.516 for Step 4 (ps < 0.0001).*

Demographic covariates explained a significant 3.5% of the variance. However, only older age (>45 years) displayed a large, significant β in the final model (model 4). This indicates a stronger association between QoL and resilience after middle age. The finding on age was consistent across all four models.

### Does Every Culture Show a Significant Positive Association Between QoL and Resilience?

When cultures were entered into the final model, only 10 out of 14 confirmed the significant positive associations that were hypothesized (Table 2, model 4). Ranked by size, the strongest positive β’s were found for Argentina, then Kenya, Uruguay, Thailand, Egypt, Malaysia, and Brazil. Weaker but significant β’s were shown by Turkey, India, and Lithuania. These 10 cultures confirmed significant positive associations across a worldwide range of cultures, with a spectrum of national income levels. Small effects and non-significant results were shown by three high-income countries – Japan, Spain, and Israel – although each country had been significant in an earlier model. The negative association found for Italy was unexpected. Although a majority of cultures confirmed the hypothesis, four did not show a significant positive association; two of these contributed fewer numbers than the target, which may have affected their result. We cannot therefore describe the relationship between resilience and QoL as “universal.”

### Which Qualities of Life Best Explain Resilience Internationally? Are Particular Qualities Linked to Defining Resilience and Practical Strategies for Building it?

International qualities of life explained a substantial 37.0% of resilience, but only six of the 13 facets showed large significant positive β’s in model 4 (Table 2). The strongest associations were found for facets on meaning in life, wholeness and integration, awe and wonder, and kindness to others (*p* < 0.0001). These four spiritual qualities form part of the SRPB domain and explained 28.2% of the total variance in resilience. Furthermore, these properties were previously linked with practical strategies for building resilience.

Two other QoL facets, indicating strong positive feelings and weak negative feelings, explained 8.8% of the variance (*p* < 0.0001) in model 4. These psychological dimensions of QoL indicate that contrasting emotions best define the resilience concept. Partial correlations (*r*) showed that good QoL from positive feelings was the most important of the two, by providing the larger contribution. However, these aspects of emotional QoL together, accounted for about one-third of the resilience explained by the four spiritual qualities combined. By introducing culture, we found that only half the qualities of life identified from previous resilience studies could be confirmed. However, these qualities could represent a parsimonious global core.

### Is the Association Between Resilience and QoL Related to Poverty?

When country β’s in the final model were inspected, a negative relationship was observed between the national poverty level and the strength of association between QoL and resilience. Using *post hoc* analysis, the national β’s from the countries in model 4 were paired with HDI values for participating countries published for the same survey year. These HDI values ranged from 0.49 in Kenya (low income) to 0.93 in Japan (high income country). The bivariate Pearson (one-tailed) correlation was negative, as expected. However, the result was small and non-significant (*r* = −0.309 (13), *p* = 0.141), showing a negligible correlation with low national income and therefore more poverty. We also observed that some wealthy countries, notably Japan, Spain, and Israel, had shown non-significant associations.

## Discussion

Information from 15 countries worldwide was analyzed to examine the impact of culture, when modeling the relationship between resilience to adversity, and QoL. These international data strongly confirmed the predicted positive association. Furthermore, it identified six qualities of life that were most salient. More importantly, culture explained a substantial 12% of the total resilience (52%), signifying that it should be systematically assessed in resilience research as it could lead to new understandings and offer a nuanced interpretation of this process.

To our knowledge, the present study is the first global model of subjective quality of life and resilience to examine data from so many cultures. The scope of this work, covering six continents, offers an original contribution to the field. These findings also provide substantial support for the relationship between culture, QoL, and resilience. The results suggest that, when building resilience, it is important to take account of cultural features as, in many cases, culture supplies important explanatory information. In the future, qualitative methods should be included in new mixed-methods research to explore the mechanisms underpinning this important association.

The present study found that not all qualities of life were pertinent to resilience. From 33 dimensions assessed by the WHOQOL SRPB, 13 salient facets were selected, from 30 that were available, after being identified through a major summary of the field that earmarked specific variables for testing. Previously, these qualities had been studied as individual variables or small groups, but the present study was able to evaluate larger numbers together as an integrated model. In the final model, only six qualities were relevant to resilience, but together they explained a substantial 37% of the total. Particularly strong were four spiritual qualities on meaning in life, awe and wonder, wholeness and integration, and kindness to others, which were previously connected to practical strategies for building resilience in the literature (American Psychological Association [APA], 2014). Meaning in life comes from affiliating with something greater than yourself (Jorgensen and Nafstad, 2004) and offers internal resources against threat. Being kind to others can “oil” the interpersonal “wheels” of society during hard times (Peterson and Parks, 2004). Experiencing a whole, integrated, and balanced life may provide feelings of being complete (Seligman et al., 2005). Awe and wonder about life, and the beauty, scale, and power of nature, heighten QoL (Kellert, 2009). These spiritual qualities could offer some insulation against adversity, and this may facilitate practical strategy building. However, we did not investigate whether the behaviors arising from these beliefs were put into action, and this awaits further research. Previous studies indicated that these four spiritual qualities are most relevant to people with strong personal and spiritual beliefs rather than those with a religious outlook (O’Connell and Skevington, 2005). These beliefs may therefore benefit strategic resilience-building among those holding a humanistic perspective, and this causal relationship deserves investigation.

Happiness and contentment has been connected with good QoL and resilience by Fredrickson (2006), so our international evidence confirms this positive outlook. However, more complex results showed that contrasting psychological dimensions of emotional QoL defined resilience. A greater contribution was made by positive feelings to the association between resilience and QoL than by negative feelings, as Fredrickson’s concept of a positivity ratio indicates (e.g., Fredrickson et al., 2003), although cultures were not explicitly included in her work. These QoL facets on emotions could facilitate a constructive mood within which practical resilience strategies are most profitably built using culturally adapted styles; new cross-cultural research should explore this.

The six spiritual and psychological qualities of life provide a psycho-spiritual backbone for a new cross-cultural model of resilience. This contrasts with previous models, which were predominantly psycho-social in style (American Psychological Association [APA], 2014). Assessing spiritual QoL was a low priority in earlier research, and evidence for these components in models was sparse and/or inconsistent. Furthermore, no evidence from our study has supported the view that social QoL was quintessential, even though two social QoL facets on social support and personal relations were tested. In addition, only two out of four psychological facets tested were included as significant, so the international evidence only weakly and partially supports a psychological model. This investigation was possible due to the availability of a highly multidimensional assessment that, due to its cross-cultural development, included an important, substantial, and highly elaborated spiritual QoL domain. Using this measure has enabled this new cultural psycho-spiritual model to be proposed.

Spiritual QoL has high cultural significance beyond Western countries and can play a prominent role in daily life (e.g., in Kenya, Brazil, and Thailand), so it has considerable face validity in these contexts. Due to the development of a comprehensive range of domains which had been affirmed as important by many cultures, it is now possible to assess spiritual QoL alongside more conventional QoL dimensions. A shortage of cross-cultural studies in the field and the slow progress in developing new cross-cultural methodologies have meant that non-Western cultures rarely contributed to shaping the concepts of resilience and QoL and their assessments. Before this study, it was not known whether the connection between resilience and QoL was considered relevant in these cultures, but the majority showed that good QoL was indeed associated with strong resilience. As four cultures did not confirm this association, we are unable to describe this relationship as “universal.” Had two of these countries provided larger samples, their results might have become significant, thus adding weight to a “universal” case. As three high-income countries showed negligible associations, this raises questions about whether richer nations do have a latent capacity to build resilience. With reference to QoL, Amartya Sen theorizes that people have a “universal” *capability* to develop resilience (Verkerk et al., 2001), and this could be activated if communities are threatened by adversity (e.g., flooding, tornados, fire, and displacement). Vulnerable communities might be assisted to activate latent capabilities to develop resilience before risks of environmental events increase.

Implementing a global resilience program will be necessary before 2030, when international projections indicate that rising temperatures could reach unprecedented levels, with concomitant effects on health and well-being (Interagency Working Group on Climate Change, 2010). With this in mind, we explored whether the association strength between QoL and resilience could be related to national poverty levels. Although a negative correlation was confirmed, the result was non-significant. Including more low-income countries could improve retesting this relationship in the future.

Modeling pinpointed a profile of pertinent QoL dimensions assessed by the WHOQOL SRPB that best predict resilience. To further validate this new cultural psycho-spiritual QoL model will require reassessing these dimensions when collecting fresh data. However, 132 items can be burdensome to answer, so the short WHOQOL SRPB BREF might plausibly be substituted in a new cross-cultural survey. This recent measure retains the breadth of contents of the long form, although each facet is assessed by one item only (Skevington et al., 2013).

At an individual level, this brief, personal, tailored integrated profile of selected QoL dimensions could identify those with high QoL and most resilience living in vulnerable communities. As resilience can develop during a lifetime’s exposure to adverse events, we expected to find that those over 45 years old had the strongest resilience. It seems plausible that, within their community, mature adults may be best equipped to adopt the leadership role needed to build resilience in their own cultural style; however, empirical verification is needed. These individuals might work with environmental and public health practitioners and health policy-makers to develop their community’s resilience. They would be well placed to shape a culturally acceptable intervention program that develops strong resilience appropriate to their community needs and possibly within a broader program of disaster preparations.

Such initiatives will need sound evaluation (Corvalan et al., 2000). Over 30 WHOQOL SRPB language versions are now available for cross-cultural research, and the manual can guide the development of new language versions for cultures living in potential disaster zones, during preparations. As a high level of equivalence exists between language versions (Bowden and Fox-Rushby, 2003), new versions could be readily compatible with others in existence.

Resilience combined inner strength with hope and optimism in the present study. While inner strength was most important, hope adds a perspective of the future, beyond an adverse event. Although hope motivates, and is recognized as an agent of change when the situation demands (Lopez et al., 2004), it was insufficient by itself to supply full resilience. Faced with a devastating environmental disaster that deprives communities of access to material and other physical resources, in some circumstances, inner strength and hope could be the sole resources available to sustain QoL as they are psychological. The present study therefore seems timely in view of recent projections about environmental disasters from climate change and epidemics.

There were several study limitations. Longitudinal data on resilience and QoL assessments before and after a specific life-threatening event would have benefited modeling and its applications and also strengthened causality. As all variables, including the five items that assessed resilience, were extracted from the same measure, there was some correlation between them. Although DV–IV correlations were small or negligible, it is likely that the model estimates of associations are inflated. An independent resilience measure should be substituted during model replication. As the World Health Organization conducted only one international WHOQOL SRPB survey, published in 2006, fresh data are now needed for an updated replication. Not every aspect of QoL related to resilience could be operationalized by the WHOQOL dimensions, so future modeling should test and extend the variable list. Indices of international poverty, psychological resilience and other resilience types, standard of living, and environmental adversity might also be included in future model developments.

## Conclusion

This global model offers unique empirical evidence to intergovernmental organizations concerned about progressing resilience-enhancing activities in communities facing hazardous environmental circumstances (e.g., UNDSR, UNEP, UNHCR, OECD, WHO, and UNDP). Time, resources, and commitment are needed to tailor culturally acceptable versions of a resilience intervention and its evaluation to local cultures. An application of this work would be designing a new intervention to develop community resilience that is adapted to the local culture, with the primary aim of instigating preparations before disasters occur (Corvalan et al., 2000). With community training, such activities could be led by resilient older community leaders who demonstrate high QoL relating to inner strength and hope and display good QoL for the six spiritual and psychological characteristics identified by this research. Such initiatives could involve collaborations with environmental, health, social, and educational professionals in a coordinated multi-disciplinary preventative approach. As promoting activities to build psychological resilience in high-risk environments is relatively inexpensive, implementing this type of intervention may produce an enduring community resource for long-term well-being.

## Members of the WHOQOL SRPB Group

The WHOQOL SRPB Group is a collaboration of researchers and consultants convened at the World Health Organization, Geneva: Mr. M. Bartos, Melbourne, Australia; Dr. S. Bonicato, La Plata, Argentina; Dr. M. Fleck, Porto Alegre, Brazil; Prof. Fang, Guangzhou, China; Prof. M. Kamel, Alexandria, Egypt; Dr. P. Chandra, Chennai, India; Dr. D. Bisht, Pondicherry, India; Dr. M. Amir, Beersheba, Israel; Dr. L. Brambilla, Rome, Italy; Dr. M. Tazaki, Tokyo, Japan; Dr. Omolo, Eldoret, Kenya; Dr. N. Gostautaite-Midttun, Vilnius, Lithuania; Dr. H. Ismail, Kubang, Malaysia; Dr. R. Lucas, Barcelona, Spain; Dr. M. Eisenmann, Umeå, Sweden; Dr. K. Meesapya, Bangkok, Thailand; Dr. H. Elbi, İzmir, Turkey; Prof. S. Skevington, Bath, United Kingdom; Dr. L. Schwartzman, Montevideo, Uruguay. At WHO Geneva: Dr, K. O’Connell, Dr. S. Saxena, and Dr. L. Underwood, United States.

## Data Availability Statement

The datasets generated for this study will not be made publicly available. At the time of the study, WHO had a policy of not releasing its data. Requests to access these datasets should be directed to Dr. Shekhar Saxena, Division of Mental Health and Substance Abuse, World Health Organization saxenas@who.int.

## Ethics Statement

The studies involving human participants were reviewed and approved by the WHO Ethics Committee for the Division of Mental Health and Substance Abuse: Reference not available. The patients/participants provided their written informed consent to participate in this study.

## Author Contributions

The WHOQOL SRPB Group centers collected their own national survey data. As an international lead in the collaboration, SS developed the research question, modeled the data, and wrote the report.

## Conflict of Interest

The authors declare that the research was conducted in the absence of any commercial or financial relationships that could be construed as a potential conflict of interest.

## Footnotes

1 http:/www.apa.org/helpcentre/road-resilience.aspx

## References

[B1] Akinyemi O. O. Owoaje E. T. Ige O. K. Popoola O. A. (2012). Comparative study of mental health and quality of life in long-term refugees and host populations in Oruljebu, Southwest Nigeria. *BMC Res. Notes* 5:394. 10.1186/1756-0500-5-394 22846111 PMC3461488

[B2] American Psychological Association [APA] (2014). *The Road to Resilience.* Washington, DC: APA.

[B3] Ardalan A. Mazaheri M. Vanrooyen M. Mowafi H. (2011). Post-disaster quality of life among older survivors 5 years after the Bam earthquake: implications for recovery policy. *Aging Soc.* 31 179–196. 10.1017/s0144686x10000772

[B4] Bottolfs M. Stea E. M. Reinboth M. S. Svendsen M. V. Schmidt S. K. Oellingrath I. M. (2020). Resilience and lifestyle - related factors as predictors for health-related quality of life among early adolescents: a cross-sectional study. *J. Intern. Med. Res.* 48 1–15. 10.1177/0300060520903656 PMC7111039 32070172

[B5] Bowden A. Fox-Rushby J. (2003). Systematic and critical review of the process of translation and adaptation of generic health-related quality of life measures in Africa, Asia, Eastern Europe, the Middle East and South America. *Soc. Sci. Med.* 7 1289–1306. 10.1016/s0277-9536(02)00503-8 12899911

[B6] Corvalan C. Briggs D. Zielhuis G. (2000). *Decision-Making in Environmental Health: From Evidence To Action.* Geneva: World Health Organisation.

[B7] Curtis S. (2004). *Health & Inequality: geographical Perspectives.* London: Sage Publications.

[B8] Fredrickson B. L. (2006). “The broaden-and-build theory of positive emotions,” in *The Science of Well-Being*, eds Huppert F. A. Baylis N. Keverne B. (Oxford: Oxford University Press), 217–240.

[B9] Fredrickson B. L. Tugade M. Waugh C. E. Larkin G. R. (2003). What good are positive emotions in crises? A prospective study of resilience and emotions following the terrorist attacks on the United States on September 11th, 2001. *J. Pers. Soc. Psychol.* 84 365–376. 10.1037/0022-3514.84.2.365 12585810 PMC2755263

[B10] Grant L. Kinman G. (2012). Enhancing wellbeing in social work students: building resilience in the next generation. *Soc. Work Educ.* 31 605–621. 10.1080/02615479.2011.590931

[B11] Guo H. X. Chen H. Teresa B. K. Chen Q. Au M. L. (2012). Factors influencing the quality of life of elderly living in a prefabricated housing complex in the Sichuan earthquake area. *J. Nurs.* 59 61–71. 22314651

[B12] Hammoudeh W. Hogan D. Giacaman R. (2013). Quality of life, human insecurity, and distress among palestinians in the Gaza strip before and after winter 2008-9 Israeli war. *Q. Life Res.* 22 2371–2379. 10.1007/s11136-013-0386-9 23479210 PMC4213857

[B13] Interagency Working Group on Climate Change (2010). *A Human Health Perspective: On Climate Change: A Report Outlining The Research Needs On The Human Health Effects Of Climate Change.* Research Triangle, CA: The National Institute of Environmental Health Sciences.

[B14] Jorgensen I. S. Nafstad H. E. (2004). “Positive psychology: historical, philosophical and epistemological perspectives,” in *Positive Psychology in Practice*, eds Linley P. A. Joseph S. (Chichester: John Wiley & Sons), 15–34.

[B15] Katz M. H. (2006). *Multivariate Analysis: A Practical Guide For Clinicians.* Cambridge: Cambridge University Press.

[B16] Kellert S. R. (2009). “Biodiversity: quality of life and evolutionary psychology,” in *Biodiversity Change And Human Health: From Ecosystem Services To Spread Of Disease. SCOPE 69*, eds Sala O. E. Meyerson L. A. Parmesan C. (Washington, DC: Island Press), 99–128.

[B17] Lercher P. (2003). Which health outcomes should be measured in health-related environmental quality of life studies? *Landsc. Urban Plann.* 65 63–72. 10.1016/s0169-2046(02)00238-4

[B18] Lopez S. J. Snyder C. R. Magyar-Moe J. L. Edwards L. M. Perdrotti J. T. Janowski K. (2004). “Strategies for accentuating hope,” in *Positive Psychology in Practice*, eds Linley P. A. Joseph S. (Chichester: John Wiley & Sons), 388–404. 10.1002/9780470939338.ch24

[B19] Manly J. J. (2006). Deconstructing race and ethnicity- implications for the measurement of health outcomes. *Med. Care* 44 S10–S16. 17060816 10.1097/01.mlr.0000245427.22788.be

[B20] O’Connell K. A. Skevington S. M. (2005). The relevance of spirituality, religion and personal beliefs to health-related quality of life: themes from focus groups in Britain. *Br. J. Health Psychol.* 10 379–398. 10.1348/135910705x25471 16238854

[B21] Pabilonia W. Combs S. P. Cook P. F. (2010). Knowledge and quality of life in female torture survivors. *Torture* 20 4–22. 20228450

[B22] Peterson C. Parks N. (2004). “Classification and measurement of character strengths: implications for practice,” in *Positive Psychology in Practice*, eds Linley P. A. Joseph S. (Chichester: John Wiley & Sons), 433–446. 10.1002/9780470939338.ch27

[B23] Rodin J. (2014). *The Resilience Dividend: Being strong in a world where things go wrong.* New York, NY: Rockefeller Foundation.

[B24] Seligman M. E. P. Parks A. C. Steen T. (2005). “A balanced psychology and a full life,” in *The Science of Well-Being*, Chap. 10, eds Huppert F. A. Baylis N. Keverne B. (Oxford: Oxford University Press).

[B25] Simonelli G. Leanza Y. Boilard A. Hyland M. Austinavicius J. L. Cardinali D. P. (2013). Sleep and the quality of life in urban poverty: the effect of slum housing upgrading program. *Sleep* 36 1669–1676. 10.5665/sleep.3124 24179300 PMC3792384

[B26] Skevington S. M. (2009). Conceptualizing dimensions of quality of life in poverty. *J. Commun. Appl. Soc. Psychol.* 19 33–50. 10.1002/casp.978

[B27] Skevington S. M. Boehnke J. M. (2018). How is subjective wellbeing related to quality of life? Do we need two constructs and both measures? *Soc. Sci. Med.* 206 22–30. 10.1016/j.socscimed.2018.04.005 29680769

[B28] Skevington S. M. Emsley R. Dehner S. Walker I. Reynolds S. E. (2019). Does subjective health affect the association between biodiversity and quality of life? Insights from international data. *Appl. Res. Q. Life* 14 1315–1331. 10.1007/s11482-018-9649-5

[B29] Skevington S. M. Epton T. (2018). How will the sustainable development goals deliver changes in wellbeing? A systematic review and meta-analysis to investigate whether WHOQOL-BREF scores respond to change. *Br. Med. J. Glob. Health* 3(Suppl. 1):e000609. 10.1136/bmjgh-2017-000609 29379649 PMC5759710

[B30] Skevington S. M. Gunson K. S. E. O’Connell K. A. (2013). Introducing the WHOQOL SRPB BREF: developing a short-form instrument for assessing quality of life in spiritual, religious and personal beliefs within quality of life. *Q. Life Res.* 22 1073–1083. 10.1007/s11136-012-0237-0 22836375

[B31] Skevington S. M. Sartorius N. Amir M., and The Whoqol Group (2004). Developing methods for assessing quality of life in different cultural settings: the history of the WHOQOL instruments. *Soc. Psychiat. Psychiatr. Epidemiol.* 39 1–8. 10.1007/s00127-004-0700-5 15022040

[B32] Skevington S. M. Tucker C. (1999). Designing response scales for cross-cultural use: data from the development of the UK WHOQOL. *Br. J. Med. Psychol.* 72 51–61. 10.1348/000711299159817 10194572

[B33] Tabachnick B. G. Fidell L. S. (2006). *Using Multivariate Statistics.* Boston, MA: Allyn & Bacon.

[B34] Ungar M. (2008). Resilience across cultures. *Br. J. Soc. Work* 38 218–235. 10.1093/bjsw/bcl343

[B35] United Nations Development Program [UNDP] (1990). *Human Development Report.* Oxford: Oxford University Press.

[B36] United Nations Environment Program [UNEP] (2011). *Health and Wellbeing Of Communities Directly Dependent On Ecosystem Goods And Services: An Indicator For The Convention On Biological Diversity.* Cambridge: UNEP.

[B37] United Nations Strategy for Disaster Reduction [UNSDR] (2009). *International Strategy for Disaster Reduction.* Geneva: UNSDR.

[B38] Valenti M. Masedu F. Mazza M. Tiberti S. Di Giovanni C. Calvarese A. (2013). A longitudinal study of quality of life of earthquake survivors in L’Aquila, Italy. *BMC Public Health* 13:1150 10.1186/1756-0500-5-1150 PMC4029473 24314066

[B39] Verkerk M. A. Busschback J. J. L. Karssing E. D. (2001). Health-related quality of life research and the capability approach of Amartya Sen. *Q. Life Res.* 10 49–55. 10.1023/a:1016652515418 11508475

[B40] Whitmee S. Haines A. Beyrer C. Boltz F. Capon A. G. deSouza Dias B. F. (2015). Safeguarding human health in the anthropocene epoch: report of the rockefeller foundation-lancet commission on planetary health. *Lancet* 386 1973–2028. 10.1016/s0140-6736(15)60901-1 26188744

[B41] WHOQOL Group (1994). “The development of the World Health Organisation quality of life assessment instrument (The WHOQOL),” in *Quality of Life Assessment: International Perspectives*, eds Orley J. Kuyken W. (Berlin: Springer-Verlag), 41–60.

[B42] WHOQOL SRPB Group (2002). *The WHOQOL SRPB User’s Manual: Scoring And Coding For The WHOQOL SRPB Field-Test Instrument. Mental Health: Evidence And Research Department Of Mental Health & Substance Abuse.* Geneva: World Health Organisation.

[B43] WHOQOL SRPB Group (2006). A cross-cultural study of spirituality, religion and personal beliefs as components of quality of life. *Soc. Sci. Med.* 62 1486–1497. 10.1016/j.socscimed.2005.08.001 16168541

[B44] World Health Organisation [WHO] (2000). *The World Health Organisation’s study into Quality Of Life And Spirituality Religiousness And Personal Beliefs: Facet Definitions And Sample Items. Report from WHOQOL Group (Internal), Department of Mental Health & Substance Abuse.* Geneva: WHO.

[B45] Yen N. P. Yang C.-C. Chang W. P. Wang J.-D. Hwang J.-S. Chang T.-C. (2013). Late effects on the health-related quality of life in a cohort population decades after environmental radiation exposure. *Intern. J. Rad. Biol.* 89 639–644. 10.3109/09553002.2013.784423 23484866

